# Immunohistochemical Biomarkers in Gastric Cancer Research and Management

**DOI:** 10.1155/2012/868645

**Published:** 2012-06-24

**Authors:** Elena Lastraioli, Maria Raffaella Romoli, Annarosa Arcangeli

**Affiliations:** Department of Experimental Pathology and Oncology, University of Florence, 50134 Florence, Italy

## Abstract

Gastric cancer still represents a major health problem, despite a decrease in its incidence in the last years. Due to the social impact of gastric cancer (GC), there is a need for novel biomarkers in order to stratify patients into appropriate screening, surveillance, or treatment programs. Although histopathology remains the most reliable and less expensive method, numerous efforts have been made searching for novel biomarkers. In recent years, several molecules have been identified and tested for their clinical relevance in GC management. In this paper, we will focus on a well-known GC marker, whose determination is mandatory in GC, HER2, a marker whose correlation with prognosis is still controversial (VEGF-A) and a quite novel, unconventional marker, the ether-à-go-go-related gene 1 (hERG1). All these proteins can be easily detected with immunohistochemistry, a technique widely used both in diagnostic and research laboratories that represents a link between surgical and molecular pathology, basic science, and clinical medicine.

## 1. Gastric Cancer

Gastric cancer (GC) still represents a major health problem, despite a decrease in its incidence in the last years [1]. According to the most recent estimates, GC accounts for 8% of the total cancer cases and for 10% of the deaths for all cancers [2]. GC is characterized by a clear geographical distribution, with over 70% of the cases occurring in developing countries. This is partly due to dietary habits as well as *Helicobacter pylori* infection prevalence. Indeed, the reasons accounting for the decreased GC incidence in most countries are related to changes in dietary habits, amelioration of food preservation, reduction in *H. pylori* chronic infection [3–5] as well as reduction in smoking [1].

**Figure 1:**
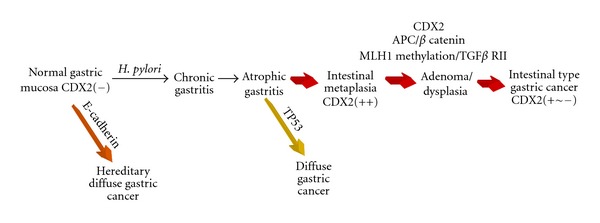
Correa model for intestinal type GC.

The majority of stomach tumors are sporadic, while only a small percentage have a familial component, with an autosomal pattern of inheritance. GC is a multifactorial disease characterized by both genetic and environmental components. In sporadic cancers of the stomach, the environmental component seems to be predominant. Conversely, the genetic component plays a major role in familial cancers. About 90% of GCs are classified as adenocarcinomas, whilst the remaining 10% is represented by non-Hodgkin lymphomas, leiomyosarcomas, squamous cell carcinomas, and undifferentiated carcinomas. In this paper, we will mainly refer to adenocarcinomas, addressing them as simply “GCs.” According to the Lauren's classification, two subtypes of GC can be distinguished basing on their different histology: the intestinal (I-GC) and diffuse (D-GC) types [6]. The two GC types also display different biological and etiological characteristics. Tumor cells of I-GC form glandular-like structures, a feature which lacks in D-GC, which, on the contrary, is characterized by the infiltration and thickening of the gastric wall by tumor cells. The two histological subtypes are the result of distinct pathogenetic pathways, well described in the two models, proposed to depict the pathogenesis of I-GC [7] and D-GC [8]. As shown in Figure 1, I-GC occurrence is preceded by the development of chronic gastritis, which in turn leads to atrophy, and by the subsequent appearance of intestinal metaplasia. Intestinal metaplasia arises from the proliferation of gastric stem cells, whose progeny differentiates into “intestinal type” cells (columnar, goblet, and Paneth cells), due to the persistent irritation of the gastric mucosa, caused by *H. pylori* [9].

**Figure 2:**
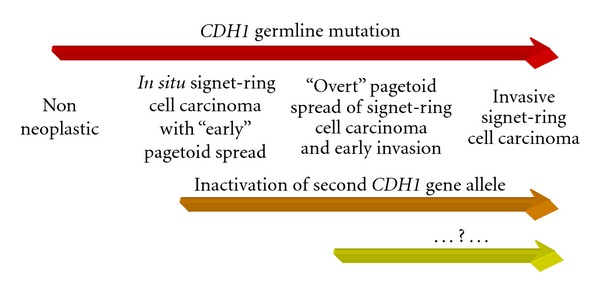
Carneiro's model for diffuse type GC.

The Correa model is not applicable to the pathogenesis of D-GC. The latter is, however, well described by the Carneiro model [8] (Figure 2).

The diffuse type GC is characterized by reduced or abnormal E-cadherin expression [10, 11]. The inactivation of the second CDH1 allele (e.g., the gene encoding E-cadherin) leads to the appearance of an *in situ* carcinoma, with the presence of signet-ring cells with a “Pagetoid” pattern of diffusion, which is subsequently followed by the invasion of surrounding tissues. According to this model, the intraepithelial presence of signet-ring cells does not represent a secondary colonization. On the whole, E-cadherin loss/abnormality represents an early event in the cancerogenesis of D-GC [8], and the dysregulation of the gene is one of the most frequent genetic alterations in diffuse type GC [12].

Due to the social impact of GC, there is a need to stratify patients into appropriate screening, surveillance, or treatment programs. Although histopathology remains the most reliable and less expensive method, numerous efforts have been made to identify and validate novel biomarkers to accomplish the above goals. In recent years, several molecules have been identified and tested for their clinical relevance in GC management.  Table 1 shows an overview of some of the biomarkers reported so far, along with the most correlated clinical parameters. With the exception of HER2, none of the biomarkers reported in the table is currently used in clinical practice, and some of them were described in single studies.

Immunohistochemistry (IHC) staining of formalin-fixed and paraffin-embedded tissues is widely used in diagnostic surgical pathology to gather additional information embracing those obtained with classical hematoxylin and eosin staining. IHC assists the pathologists in areas of tumor classification, multilineage differentiation, molecular correlates, and infectious etiologies. Moreover, IHC is commonly used to detect markers, which in turn can provide information on the biological behaviour and prognosis of a tumor. Different biomarkers detected by IHC are now a common component for many institutional review board protocols, for a more precise risk stratification and target identification. Therefore, IHC represents a link between surgical and molecular pathology, basic science and clinical medicine, surgery, and radiology [13].

As evidenced in Table 1, the number of potential biomarkers in GC is quite high and still increasing. Those which are easily detectable and quantifiable through IHC will be the object of the present review. In particular, we will focus on HER2, a well-known GC marker, whose determination is mandatory in GC, a marker whose correlation with prognosis is still controversial, VEGFs and quite novel, unconventional marker, hERG1.

### 1.1. HER2

**Figure 3:**
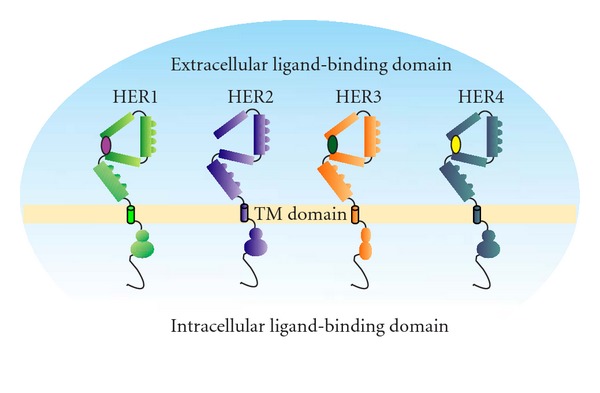
HER family receptors.

The HER family comprises four different receptors: HER1 (EGFR or ErbB1), HER2 (ErbB2 or HER-2/Neu), HER3 (ErbB3), and HER4 (ErbB4) (Figure 3).

These receptors cooperate in the regulation of different processes, such as cell proliferation, differentiation, and survival [14]. HER family members are implicated in the development of different kinds of tumors and are now recognized targets for biological therapy in breast, colorectal, lung, head and neck, gastric and gastro-oesophageal junction cancer (reviewed in [15]). Upon ligand binding, the receptors dimerize, become phosphorylated, and transduce intracellular signals, that ultimately regulate the above-mentioned cellular processes. Receptor dimerization can also occur through the process of receptor pairing, other than ligand binding [16]. Indeed, HER receptors can either homo- or hetero-dimerize with other HER family members, allowing multiple receptor combinations [16, 17].

Dimer formation leads to the phosphorylation of key intracellular proteins, that provide docking sites for a variety of subsiding signalling molecules. The latter then transmit signals to different downstream cascades, including the MAPK and the PI3K/AKT pathways [16, 18].

The HER2 gene has been recognized as a key regulator in the development of different types of tumors in particular breast cancer [60]. In GC, HER2 acts as an oncogene, since gene amplification reflects in protein overexpression, therefore giving selective advantage to malignant cells. In GC, HER2 overexpression has been correlated with poor outcome and a more aggressive disease [20] as well as with shorter survival [19–22, 61–65]. Based on data presented at the ASCO meeting in 2009, about 22% of patients with advanced GC have tumors which overexpress HER2. In a large multicentric trial carried out on GC patients (ToGA study), a survival benefit of trastuzumab (herceptin) treatment in HER2-positive patients (IHC score 3+) has been shown [23]. Therefore, HER2 represents a promising therapeutic target. However the optimal HER2 testing strategy has not been defined yet. Due to the recent approval of trastuzumab for HER2-positive GC in Europe, HER2 diagnostics is now mandatory: IHC is used as primary test, and it is followed by fluorescence *in situ* hybridization (FISH) in IHC2+ cases [27]. A more recent paper [26] showed that HER2 amplification can be detected in the two components (intestinal type and diffuse type areas of the neoplastic lesion) of Lauren mixed-type tumors. Standardization of HER2 testing procedures and interpretation is, therefore, an essential step to ensure accurate and reproducible results. This point acquires even more relevance, since it has been shown that HER2 status determination through the same protocol used for breast cancer, might lead to significant loss of patients [66] as there are important and significant differences in HER2 status determination between the two types of cancer (Table 2, see also [67]). As reported in Table 2, samples are given a score according to the intensity, degree of membrane reactivity and the percentage of immunoreactive cells. Scores 0 and 1+ are considered as negative, score 3+ is considered as positive, while 2+ samples are considered as equivocal and should be retested by fluorescence *in situ* hybridization (FISH) and chromogenic *in situ* hybridization (CISH).

If FISH is used as first screening step, only few IHC3+ cases may be missed but it might be found a high percentage of nonresponders according to ToGA results [24, 68]. In a more recent study published in 2011 [69] it was shown that HER2 testing in GC could be performed using standard breast cancer procedures and the American Society of Clinical Oncology/College of American Pathologists scoring criteria, while a group from Korea concluded that a GC-specific scoring system should be used [70].

In contrast to HER2 and despite supportive preclinical data, observed clinical success with anti-HER1 inhibitors and endocrine therapy combinations in breast cancer has been limited [71, 72]. In addition to HER1 and HER2, there is growing interest in HER3 as a potential therapeutic target [73]. Recently, HER3 and its physiologic ligand heregulin (HRG) have been implicated in the development of resistance to antiestrogen therapies in breast carcinoma [74]. Similarly, the dual HER1 and HER2 TKI lapatinib has clinical activity and is approved for the therapy of patients whose disease has progressed on trastuzumab [74]. Pertuzumab is a recombinant humanized monoclonal antibody directed against the dimerization domain II of HER2 that is required for ligand-dependent dimerization with HER3 [74]. While trastuzumab prevents ligand-independent HER2 signaling, pertuzumab interferes with ligand-dependent HER3-mediated signaling.

### 1.2. VEGF

**Figure 4:**
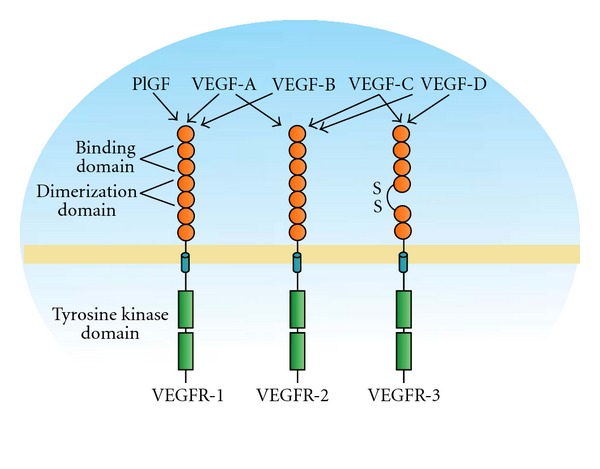
VEGF receptors and their ligands.

The vascular endothelial growth factor (VEGF) family is a multifunctional growth factors' family, involved in processes such as angiogenesis, inflammation, and vascular regeneration. The family includes different members: VEGF-A, VEGF-B, VEGF-C, VEGF-D, VEGF-E, and PlGF, characterized by the different ability to bind to three main receptors Flt1, KDR, and Flt4 (Figure 4).

It has long been known that VEGF-A is the key regulator of tumor angiogenesis [75], a complex process with a clear relevance to tumor progression and metastasis. The maximum diameter a tumor can reach without developing a new vascular network is about 1-2 mm. Hypoxia within tumor mass induces VEGF-A secretion and increased VEGFR-2 expression [76].

It has been demonstrated that VEGF-A expression is higher in I-GC compared to D-GC [32]. The expression of VEGF-C, whose main role is that of promoting lymphangiogenesis, is related to lymph node metastasis in GC [77]. VEGF expression is mirrored by microvessel density (MVD), and they are both hallmarks of enhanced angiogenesis within the tumor mass and are therefore useful tools for GC management [78]. MVD has been investigated as a promoting factor for angiogenesis with conflicting results about its relation to survival in GC. VEGF secretion promotes endothelial cell proliferation and therefore the establishment of a new vascular network. The evaluation of MVD reflects this latter process since it is evaluated by IHC with anti-CD34 or anti-CD31 antibodies which specifically indicate new formed vessels. MVD was significantly related to the T stage, as to the TNM classification, while VEGF-C expression was significantly higher in N-positive patients [79]. No relation was found between MVD and VEGF-C expression, but VEGF-C and MVD turned out to be related to clinicopathological features [79].

Although the VEGF superfamily has been identified to critically influence tumor-related angiogenesis, the prognostic significance of VEGF expression in GC is still controversial. In particular, VEGF-A expression seems to be a negative prognostic factor, at least in EGCs [80]. Moreover, VEGF-A expression has been proven to be relevant to therapeutic response in GC patients treated with fluorouracil alone or together with cisplatin [81]. We contributed to this discussion showing that the IHC expression of VEGF-A, which positively correlated with the Lauren's intestinal histotype, has a positive impact on overall survival in univariate analysis (manuscript in preparation). The reasons of the different conclusions drawn by several groups might be related to the design of the study and sample characteristics as well as geographical differences, keeping in mind that GC is a complex disease with striking differences in different countries. Furthermore, a recent paper [33], in which the impact of VEGF-A/C/D on tumor dissemination and survival in GC was evaluated, led to conclude that VEGF-D, being associated with progressive disease, could be a helpful marker of disseminated disease. The authors concluded that the targeting of VEGF-D might be therefore a potential therapeutic strategy.

Besides controversies in the interpretation of IHC data, the Avastin in Gastric Cancer (AVAGAST) trial started in 2007. It was a multinational, randomized, and placebo-controlled trial aimed to evaluate the efficacy of adding bevacizumab to capecitabine-cisplatin in the first-line treatment of advanced gastric cancer. Although AVAGAST did not reach its primary objective, adding bevacizumab to chemotherapy was associated with significant increases in progression-free survival and overall response rate in the first-line treatment of advanced gastric cancer [34].

### 1.3. hERG1

**Figure 5:**
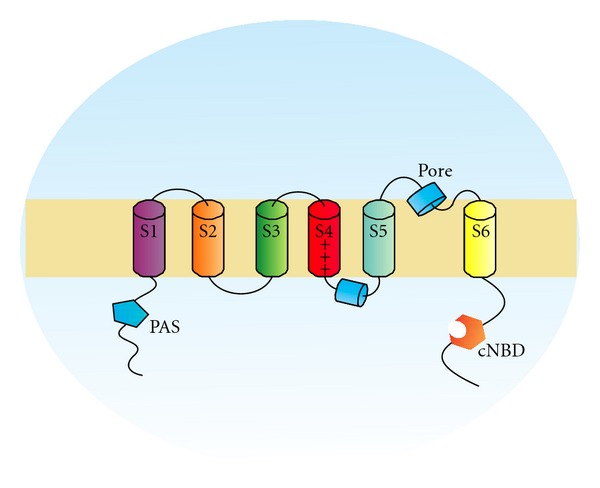
hERG1 potassium channel structure; PAS: Per Arnt Sim domain, cNBD: cyclic nucleotide-binding domain.

The *human ether-à-go-go*-*related gene 1* (*hERG1*) encodes for a protein, hERG1, which is functionally a voltage-dependent potassium channel (K_V_), with outward rectifying characteristics. hERG1 has the typical structure of K_V_s: it is composed of four subunits, each of which formed by six transmembrane segments (S1–S6), which are assembled to form a tetramer surrounding a central aqueous pore. The S4 segment of each subunit is composed of basic aminoacids (Lys and Arg) and represents the voltage sensor [82] (Figure 5).

hERG1 constitutes the molecular basis of the cardiac rapid repolarizing current (IKr) (reviewed in [83]) and is therefore physiologically relevant to regulate the cardiac action potential [83]. In addition, hERG1 was found to be over- and mis-expressed in a wide variety of human cancers [84–93], where its activity is relevant to drive tumor progression. In particular, hERG1 activity is modulated by hypoxia [94] and regulates VEGF-A secretion in astrocytomas [84]. In addition, hERG1 channels mediate VEGF-Receptor-1 (FLT-1)-induced cell migration and signalling in acute myeloid leukemias [90]. hERG1 is also overexpressed in cancers of the gastrointestinal tract, in particular in colorectal [91, 92] and oesophageal adenocarcinomas [93]. In colorectal cancer, it has been recently demonstrated that the IHC positivity to hERG1, in conjunction to lack of expression of the glucose transporter 1 (Glut-1) is an independent negative prognostic factor in TNM stages I and II colorectal cancers [91].

A few papers addressing the expression and role of hERG1 in GC have been published so far. hERG1 channels are expressed in GC cell lines, where their activity regulates cell proliferation *in vitro* [95, 96]. Shao and colleagues [95] demonstrated that cisapride, a specific blocker of hERG1, can inhibit the growth of GC cells, by altering cell distribution within the cell cycle and inducing apoptosis. A more recent paper by the same group [97] demonstrated the correlation between hERG1 expression and tumor grading and TNM stage. Furthermore, inhibition of the channel with specific siRNAs resulted in a reduction of tumor growth and colony formation. Based on these results, hERG1 protein could be considered as a potential therapeutic target. More recently, Ding and colleagues [35] demonstrated significant differences in hERG1 protein expression, according to factors such as serosal invasion, venous invasion, and TNM stage. The mean survival time for hERG1 positive patients was significantly shorter than that of hERG1 negative ones and hERG1 expression was proven to be an independent prognostic factor [35]. To our knowledge, these are the only available data concerning hERG1 in GC addressing hERG1 as a negative prognostic factor. On the contrary when performing a study in a larger cohort of 524 GC patients (manuscript in preparation) encompassing different stages of the disease, we obtained different conclusions. In particular, our data confirmed that hERG1 is an independent prognostic factor, but we demonstrated its association with positive prognosis.

## 2. Concluding Remarks

Histopathology still represents the most powerful tool for gastric cancer management and, in recent years, novel biomarkers have been identified and tested for their correlations with clinical parameters as well as prognosis. In the near future new markers will be certainly validated, and the use of genomics and proteomics might help greatly clinicians in cancer management. Anyway, the possibility of validating potential tumor markers using IHC has clear advantages as it is easy and cost effective and virtually every pathology laboratory could perform it. Taking into account the importance and the usefulness of IHC markers, it will be of great importance in the next future to keep on searching for novel biomarkers as well as validating those already identified.

## Figures and Tables

**Table 1:** Immunohistochemical markers in GC.

IHC marker	Parameter	Reference
HER2	Prognosis	[19–23]
	Therapeutic response	[24]
	Lymph node metastasis	[25]
	Lauren histotype	[26, 27]

VEGF	Prognosis	[28–31]
	Lauren histotype	[32]
	Tumor progression	[33]
	Therapeutic response	[34]

hERG1	Prognosis	[35]

KLF5	Grading	[36]
	Stage	[36, 37]
	Lymph node status	[36, 37]
	Prognosis	[36, 37]

CA IX	Lymph node metastasis	[38]
	Prognosis	[38]

Ki67	Lymph node metastasis	[25]

PKP3	Stage	[39]
	Prognosis	[39]

MMP-2	Prognosis	[29, 31]
HDAC	Prognosis	[40–42]
*Bcl-2*	Lymph node metastasis	[25]
* Bcl-6*	Prognosis	[43]

*SATB1*	Lymph node metastasis	[44, 45]
	Distant metastasis	[44, 45]
	Stage	[44, 45]

*c-myc2*	Lymph node metastasis	[25]
TGF *β*	Stage	[46]

E-cadherin	Prognosis	[31, 47–52]
	Invasion	[53]
	Grading	[54, 55]
	Lauren histotype	[54]

COX-2	Prognosis	[56]
TSP-1	Prognosis	[57]
Bax	Prognosis	[58, 59]

**Table 2:** HER2 testing by immunohistochemistry in gastric cancer.

IHC parameters for HER2 testing protocol	IHC score	Classification
Intensity of reactivity		
Absent	0	Negative
Faint	1+	Negative
Weak to moderate	2+	Equivocal^∗^
Moderate to strong	3+	Positive

Degree of membrane reactivity		
Complete	2+	Equivocal^∗^
	3+	Positive
Incomplete	0	Negative
	1+	Negative

Percentage of immunoreactive cells (membrane reactivity)		
≥10%	1+	Negative
	2+	Equivocal^∗^
	3+	Positive
<10%	0	Negative

^∗^ Samples scored IHC 2+ should be retested with fluorescence *in situ* hybridization (FISH) or chromogenic *in situ* hybridization (CISH).
